# Effect of High Glucose and Carboxymethyl-Lysine on Osteocyte Gene Expression

**DOI:** 10.4236/ajmb.2025.152012

**Published:** 2025-03-21

**Authors:** Rachana Vaidya, Lauren Conlon, Olivia Duclos, Ramina Behzad, Jacob Aaronson, Lamya Karim

**Affiliations:** 1 Department of Bioengineering, University of Massachusetts Dartmouth, Dartmouth, MA, USA; 2 Department of Orthopaedic Surgery, Washington University School of Medicine, St. Louis, MO, USA

**Keywords:** Osteocytes, Advanced Glycation End Products (AGEs), Diabetes Mellitus, Bone Fragility, Inflammation, Glycation

## Abstract

Diabetes mellitus (DM) is associated with increased bone fragility despite normal or elevated bone mineral density, partially due to the accumulation of advanced glycation end products (AGEs) in bone tissue. AGEs, such as carboxymethyl lysine (CML), impair osteocyte function by activating the receptor for advanced glycation end products (RAGE), triggering oxidative stress and inflammatory responses. This study aimed to investigate the effects of high glucose (HG) and CML on bone remodeling, glycation, inflammatory markers, and cellular functions in osteocytes. Using the murine osteocyte cell line OCY454-12H, we treated cells with HG (30 mM glucose) or 3 μM CML to simulate diabetic conditions. We assessed the expression of bone remodeling markers (SOST, RANKL, OPG, CTsK), glycation markers (RAGE, AGER1), inflammatory cytokines (IL-6, TNF-*α*), and cellular functions, including proliferation, viability, and apoptosis, using quantitative PCR and functional assays. HG treatment resulted in a 10-fold increase in SOST expression (9.3 vs. 0.9, p ≤ 0.0001) and a 2.4-fold increase in RANKL expression (2.75 vs. 1.15, p ≤ 0.0001), with a concurrent 2-fold increase in OPG (2.60 vs. 1.04, p ≤ 0.0001). The RANKL/OPG ratio remained unchanged (p = 0.15). HG also significantly increased RAGE expression by 3.67-fold (4.20 vs. 1.15, p ≤ 0.0001) and AGER1 by 1.65-fold (1.94 vs. 1.15, p ≤ 0.0001), along with a 2.02-fold increase in IL-6 (2.32 vs. 1.12, p ≤ 0.001) and a 7.35-fold increase in TNF-*α* (7.04 vs. 1.04, p ≤ 0.0001). Cell viability and proliferation were significantly higher under HG, accompanied by increased caspase-3 activity, indicating enhanced apoptosis. In contrast, CML exposure significantly upregulated RAGE (3.18 vs. 1.15, p ≤ 0.0001) and AGER1 (2.10 vs. 1.14, p = 0.028) but had no significant effects on bone remodeling markers, inflammatory cytokines, or cellular functions at physiological concentrations. Our findings demonstrate that HG disrupts osteocyte function by altering bone remodeling, glycation, and inflammatory pathways, while CML at physiological levels selectively activates glycation markers without inducing broader cellular dysfunction. These results underscore the role of the AGE-RAGE axis in diabetic bone fragility and highlight the need for future *in vivo* studies to explore therapeutic strategies targeting AGE accumulation and RAGE signaling in bone.

## 1. Introduction

Diabetes mellitus (DM) is a chronic metabolic disorder characterized by persistent hyperglycemia, which has profound systemic consequences, including an increased risk of skeletal fragility [1] [2]. Despite normal or even elevated bone mineral density observed in individuals with diabetes, their susceptibility to fractures is markedly increased, indicating compromised bone quality [3]-[5]. The bone mineral density discordant fracture prevalence is largely attributed to alterations in bone microarchitecture, reduced bone remodeling, and the accumulation of advanced glycation end products (AGEs) in the bone matrix [6]-[10].

AGEs are formed through non-enzymatic glycation reactions driven by prolonged hyperglycemia [11]. Among the various AGEs, carboxymethyl-lysine (CML) is one of the most extensively studied, particularly in the context of diabetes. CML is commonly detected in tissues of individuals with diabetes and is associated with increased oxidative stress [12]-[14], reduced insulin sensitivity [15], and impaired renal function [11] [16]. Dietary modulation of CML has been shown to influence systemic health outcomes significantly, with diets low in CML associated with lower insulin resistance, visceral fat, and oxidative stress [17] [18], whereas high-CML diets promote insulin resistance and inflammation [19] [20], largely mediated through the receptor for AGEs (RAGE) [21]-[23]. Given its significant impact on inflammation and metabolic regulation, CML has been investigated as a potential biomarker for diabetes-associated skeletal complications. However, further studies are needed to establish its clinical utility [24] [25].

Recent evidence suggests that hyperglycemia may directly impact the extracellular matrix of the bone, particularly its composition and organization, ultimately weakening bone integrity [26]. It is also well documented that CML and other AGEs can impair the biomechanical properties of the bone matrix, increasing its brittleness and susceptibility to fracture through non-enzymatic glycation of collagen and other structural proteins in the bone extracellular matrix [27]-[31]. Additionally, AGEs can bind to their specific receptor, the receptor for advanced glycation end products (RAGE), triggering a cascade of signaling pathways that promote oxidative stress and inflammation [32] [33]. In diabetes, the chronic activation of RAGE signaling in diabetes disrupts the balance of bone remodeling, by suppressing osteoblast-mediated bone formation and enhancing osteoclast-mediated bone resorption [34]-[39], further compromising bone integrity. Among bone cells, osteocytes are particularly vulnerable to AGE-RAGE interactions, as this signaling cascade impairs their regulatory function in maintaining skeletal homeostasis [26]. However, cellular defense mechanisms exist to counteract AGE-induced damage, notably through the advanced glycation end product receptor 1 (AGER1), which promotes AGE detoxification and suppresses RAGE-mediated inflammatory responses [40] [41]. Understanding the balance between RAGE activation and AGER1 expression is important to elucidating the molecular pathways contributing to diabetes-induced skeletal fragility.

Osteocytes, the most abundant and long-lived cells within the bone matrix [42] [43], play a pivotal role in maintaining skeletal homeostasis [44]. These cells act as mechanosensors and orchestrate bone remodeling by regulating the activity of osteoblasts and osteoclasts [45]. Osteocytes can promote bone resorption by expressing receptor activator of nuclear factor kappa-B ligand (RANKL) [46] [47] and cathepsin K (CTsK) [48] [49], key molecules involved in osteoclast differentiation and function. Conversely, osteocytes inhibit bone formation by secreting sclerostin (SOST) [50] [51], which has been found to be elevated in T2D patients [52]-[56]. Osteocytes also secrete osteoprotegerin (OPG) [57]-[59], a decoy receptor for RANKL, providing a mechanism to balance bone resorption and formation. Emerging evidence suggests that osteocytes are particularly vulnerable to the deleterious effects of AGEs and hyperglycemia [54] [60]-[62]. Unlike osteoblasts and osteoclasts, osteocytes are embedded within the mineralized matrix and have limited capacity for renewal, making them more susceptible to AGE accumulation over time. The accumulation of AGEs and activation of RAGE signaling in osteocytes may disrupt their function, contribute to impaired regulation of bone remodeling and alter the lacuno-canalicular network [63]. These disruptions could compromise bone quality by reducing bone turnover, increasing cortical porosity, and weakening bone microarchitecture, ultimately contributing to the increased fracture risk observed in diabetic patients. Thus, osteocyte dysfunction may play a crucial role in diabetic bone fragility by impairing the fine balance between bone resorption and formation, despite normal or elevated bone mineral density in T2D. However, the specific mechanisms through which hyperglycemia and CML influence osteocyte function are not well understood. Given that osteocytes are responsible for orchestrating bone remodeling in response to metabolic and mechanical signals, understanding their response to hyperglycemic conditions is crucial to elucidating the mechanisms underlying diabetes-related bone fragility. In addition to markers of bone remodeling, inflammation plays a key role in diabetes-related skeletal alterations. Elevated levels of pro-inflammatory cytokines such as interleukin-6 (IL-6) and tumor necrosis factor-*α* (TNF-*α*) are associated with T2D [64] [65] and contribute to impaired bone turnover and increased bone resorption [66] [67]. However, the regulation of these inflammatory markers in osteocytes under hyperglycemic conditions remains poorly understood.

To address these gaps, our study employed the OCY454-12H murine osteocyte cell line to simulate and examine the cellular responses to hyperglycemia and CML exposure. Utilizing quantitative PCR, we quantitatively assessed alterations in gene expression of crucial bone remodeling, glycation, and inflammatory markers—SOST, RANKL, OPG, CTsK, RAGE, AGER1, IL-6, and TNF-*α*. This approach allowed for precise measurement of mRNA levels, providing insights into the transcriptional changes induced under diabetic conditions, which are critical for understanding the cellular pathways affected. This investigation aimed to elucidate the molecular and cellular mechanisms impacted by AGEs and RAGE signaling in osteocytes under diabetic conditions, contributing to our understanding of diabetes-induced skeletal fragility.

## 2. Methods

### 2.1. OCY454-12H Cell Culture

Conditionally immortalized Ocy454-12H osteocytic cells in this study were kindly provided by Dr. Paola Divieti Pajevic (Center for Skeletal Research at Massachusetts General Hospital, Boston, MA). Cells were maintained on type I collagen-coated flasks (Corning, #356485) in alpha-MEM (Gibco, #12571) supplemented with 10% Fetal Bovine Serum (Gibco, #26140) and 1% Antibiotic-Antimycotic 100X (Gibco, #15240) and 25mM HEPES (Sigma Aldrich, #H3375) to maintain pH. Cells were maintained in collagen-coated T-75 flasks or standard tissue culture flasks at 33°C, with media changes every 2 - 3 days. Cells were passaged every three days when reaching 90% confluence to prevent overgrowth, as over-confluence results in reduced SOST/sclerostin expression.

**Figure 1.**
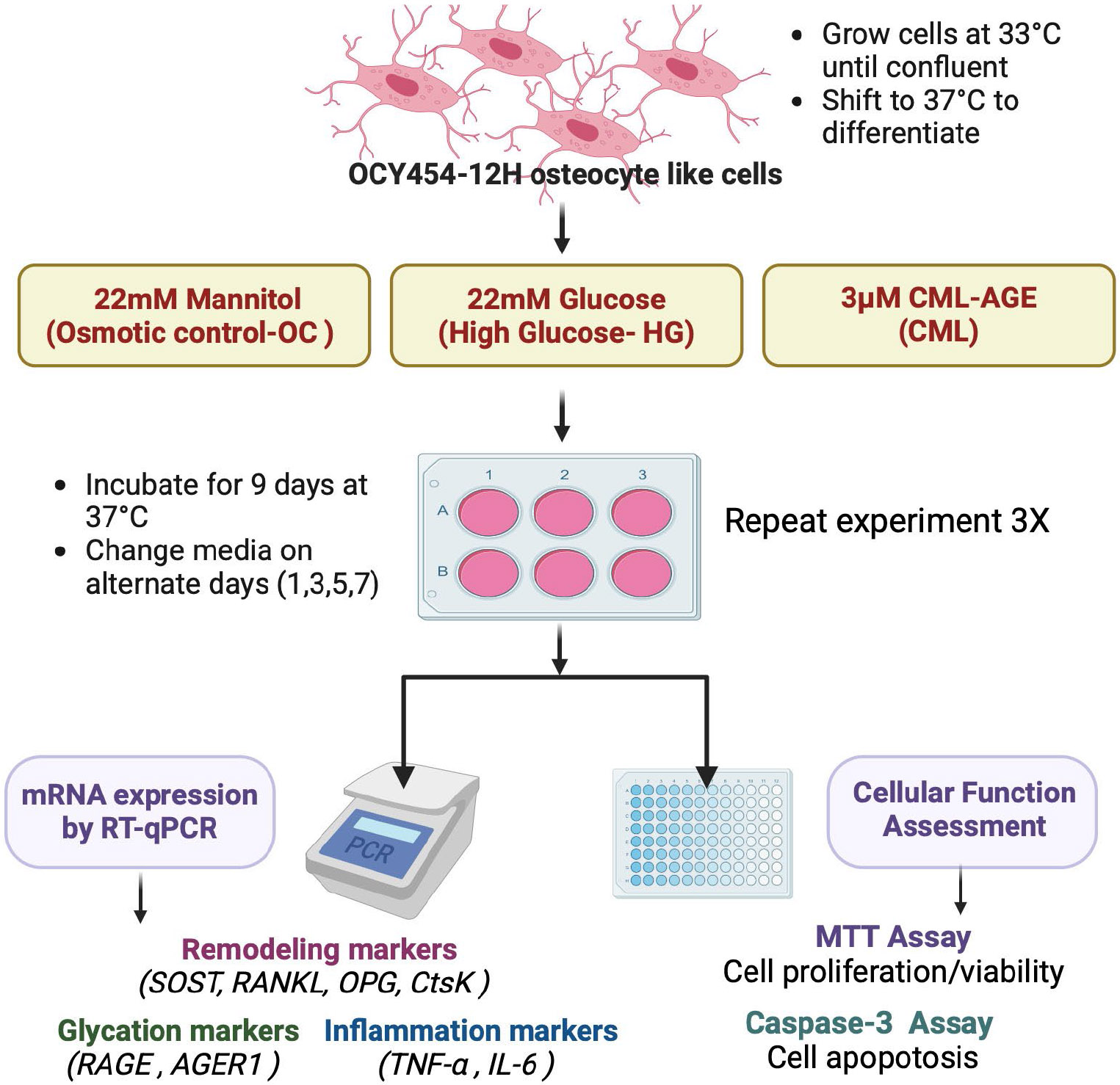
Experimental design to study the effects of high glucose and CML-AGE on OCY454 osteocyte-like cells: OCY454-12H osteocyte-like cells were cultured at 33°C until confluent and then differentiated by shifting the temperature to 37°C. Cells were treated with one of three conditions: 22 mM mannitol (osmotic control), 22 mM glucose (high glucose, HG), or 3 μM carboxymethyl lysine-advanced glycation end product (CML-AGE). Treatments were maintained for 9 days, with media changes every other day (days 1, 3, 5, and 7). The experiment was repeated three times. Following treatment, mRNA expression was analyzed by RT-qPCR to assess remodeling markers (SOST, RANKL, OPG, CtsK), glycation markers (RAGE, AGER1), and inflammation markers (TNF-*α*, IL-6). Cellular function was evaluated using the MTT assay to measure cell proliferation/viability and the Caspase-3 assay to assess cell apoptosis.

For experimental conditions, cells were plated at a density of 100,000 cells/mL in non-collagen-coated tissue culture plates. After three days, the media were replaced, and cultures were transferred to 37°C for osteocytic differentiation. The OCY454-12H cell line was specifically designed to differentiate into a mature osteocyte-like phenotype upon the inactivation of the temperature-sensitive SV40 large T antigen at 37°C. This temperature shift induces the transition from a proliferative state to a more mature osteocyte-like state, characterized by increased expression of osteocyte marker such as sclerostin. Media were subsequently changed every 2 - 3 days. Cells were maintained under these conditions for 10 - 12 days, which is the routine laboratory timepoint for achieving an osteocytic phenotype. SOST expression at this timepoint is typically between 28 - 32 Ct, with beta-actin as the reference gene at 16 - 18 Ct, as determined by RT-PCR (Figure 1).

### 2.2. High Glucose Treatment (22 mM Glucose)

For the high glucose study, OCY454 cells were grown to confluence at 33°C (approximately 2 days) and subsequently plated in six-well plates (Corning, #3516) at a density of 1 × 10^5^ cells/mL. Cells were assigned to one of three treatment groups: Standard Media Control (alpha-MEM + 5.56 mM glucose), Osmotic Control (alpha-MEM + 5.56 mM glucose supplemented with 22 mM D-mannitol), and High Glucose Media (alpha-MEM + 22 mM glucose) (Figure 1). D-glucose or D-mannitol was added to the standard media to achieve the final concentration of 22 mM glucose or mannitol, respectively. The 22 mM concentration was selected based on prior studies to model chronic hyperglycemia. This concentration ensures a reproducible diabetes environment whole maintaining cell viability over prolonged culture periods [9].

Cells were maintained at 33°C until they reached confluence and were then transferred to 37°C for differentiation. At Day 9 post-transfer to 37°C, cell lysates and culture media were collected for analysis of gene expression and marker changes in differentiated cells.

### 2.3. Carboxy-Methyl Lysine (CML-AGE) Treatment

To determine the effect of the CML on osteocytes, OCY454 cells were cultured to confluence at 33°C (2 - 3 days) and subsequently plated in six-well plates (Corning, #3516) at a density of 1 × 10^5^ cells/mL. Cells were divided into three treatment groups: Standard Media Control (alpha-MEM + 3 μM PBS), and CML-AGE (alpha-MEM + 3 μM CML). A 4.9 mM CML stock solution was prepared by dissolving 1 mg CML (Cayman, #16483) in 1 mL PBS (pH 7.2). This stock solution was diluted with the appropriate media to achieve a final concentration of 3 μM CML. Due to the instability of CML in aqueous solution beyond 24 hours, fresh media were prepared with a new CML vial for every media change, performed every alternate day. To mimic the elevated levels of carboxymethyl-lysine (CML) observed in diabetic patients, which have been reported to be 400 - 700 ng/ml (2 - 4 μM), we used a concentration of 3 μM CML in our osteocyte cell culture study to understand if this concentration could influence cell activity and bone remodeling *invitro* [68]. This dose was chosen to reflect a relevant pathological range while avoiding excessive cytotoxicity, allowing us to investigate CML-mediated effects on osteocytes under conditions that mimic diabetic bone pathology. Cells were maintained at 33°C until they reached confluence and were then transferred to 37°C for the differentiation phase. At Day 9 post-transfer to 37°C, cell lysates and culture media were collected for analysis of gene expression and other marker changes in differentiated cells.

### 2.4. mRNA Quantification with RT-qPCR

Total RNA was extracted from cell lysates using the RNeasy Plus Mini Kit (Qiagen, Hilden, Germany) according to the manufacturer’s instructions. RNA concentration and purity were assessed using a NanoDrop One Microvolume Spectrophotometer (Thermo Scientific, Waltham, MA), ensuring A260/A280 and A260/A230 ratios within acceptable ranges (≥1.8 ≤2.1). Complementary DNA (cDNA) was synthesized using the PrimeScript RT Reagent Kit with gDNA Eraser (Takara Bio Inc., Kusatsu, Japan), with 1 μg of total RNA per reaction, following the kit protocol.

Quantitative real-time polymerase chain reaction (qPCR) was performed on a QuantStudio 3 Real-Time PCR System (Applied Biosystems, Foster City, CA). Re-actions were set up using cDNA, SYBR Green PCR Master Mix (Qiagen, #204141), and specific primers for target and housekeeping genes (Integrated DNA Technologies, Coralville, IA), with a total volume of 2 μL. *β*-Actin and 18S rRNA were used as housekeeping genes to normalize gene expression.

Relative gene expression was calculated using the ΔΔCT method (2^−ΔΔCT^). ΔCT values were computed within each sample relative to the housekeeping genes, and ΔΔCT values were determined across treated and untreated control samples. All experiments were performed in triplicates, and each sample was run in technical triplicates (n = 9). Gene expression data were normalized to *β*-actin, and fold changes in expression were determined using the Livak method.

### 2.5. Cell Proliferation Measurement by MTT Assay

Cells were seeded at a density of 1 × 10^3^ cells/mL in a 96-well plate, with 100 μL of cell suspension added per well. Media without cells served as blanks for background subtraction. After incubation at 33°C for 24 hours, cells were shifted to 37°C for 5 days to promote differentiation. Media changes were performed every alternate day.

On Days 1, 3, and 5, cell proliferation was assessed. For each timepoint, the media were aspirated, and 50 μL of fresh media and 50 μL of MTT solution (5 mg/mL in PBS) were added to each well. Plates were incubated at 37°C for 3 hours, after which 150 μL of MTT solvent (DMSO) was added to dissolve the formazan crystals. The plates were covered in foil and placed on an orbital shaker for 15 minutes to ensure complete solubilization. Absorbance was measured at 590 nm using a Synergy HTX Microplate Reader (BioTek Instruments, Winooski, VT). Data were normalized to media-only blanks, and proliferation rates were calculated as fold changes relative to Day 1 (Figure 1).

### 2.6. Apoptosis Assay

Cells were plated in six-well plates as described above and allowed to differentiate for 9 days at 37°C. On Day 9, cells were harvested by adding 250 μL of TrypLE Express (Thermo Fisher Scientific) per well and incubating at 37°C for 5 minutes to detach the cells. Detached cells were resuspended in 1.25 mL control media, transferred to microcentrifuge tubes, and centrifuged at 14,000 × g for 15 minutes to pellet the cells. The supernatant was aspirated, and cell pellets were stored at −80°C until analysis. Total protein was quantified using Bradford’s assay.

Apoptosis was measured using the EnzChek Caspase-3 Activity Kit (Molecular Probes, Eugene, OR, USA) following the manufacturer’s protocol. Briefly, cell lysates were incubated with the caspase-3 substrate in a reaction buffer, and fluorescence intensity was measured at the specified excitation/emission wavelengths using a microplate reader. Positive (e.g., apoptosis inducer-treated) and negative controls were included to validate assay specificity. Data were expressed as relative caspase-3 activity per mg of protein and compared to untreated controls (Figure 1).

### 2.7. Statistical Analysis

All data are presented as mean ± standard deviation (SD) of measurements from independently performed experiments. Each experiment included three technical replicates per condition.

For comparisons between the osmotic control (OC) and individual treatment groups (high glucose HG, CML, and HG + CML), a one-way ANOVA followed by Tukey’s post hoc test was used to assess differences in gene and protein expression across the groups. When comparing two timepoints within the high glucose treatment group, a two-way ANOVA was employed to account for both treatment and time factors.

The significance level (*α*) was set at 0.05 for all analyses. Statistical analyses were performed using GraphPad Prism 9, and assumptions of normality and homogeneity of variance were verified before conducting parametric tests. If assumptions were violated, appropriate non-parametric alternatives (e.g., Kruskal-Wallis test with Dunn’s post hoc test) were applied.

## 3. Results

### 3.1. High Glucose Treatment Inhibits Bone Formation, Upregulates Glycation and Inflammatory Markers and Affects Cell Metabolism and Apoptosis in Osteocytes

**Figure 2.**
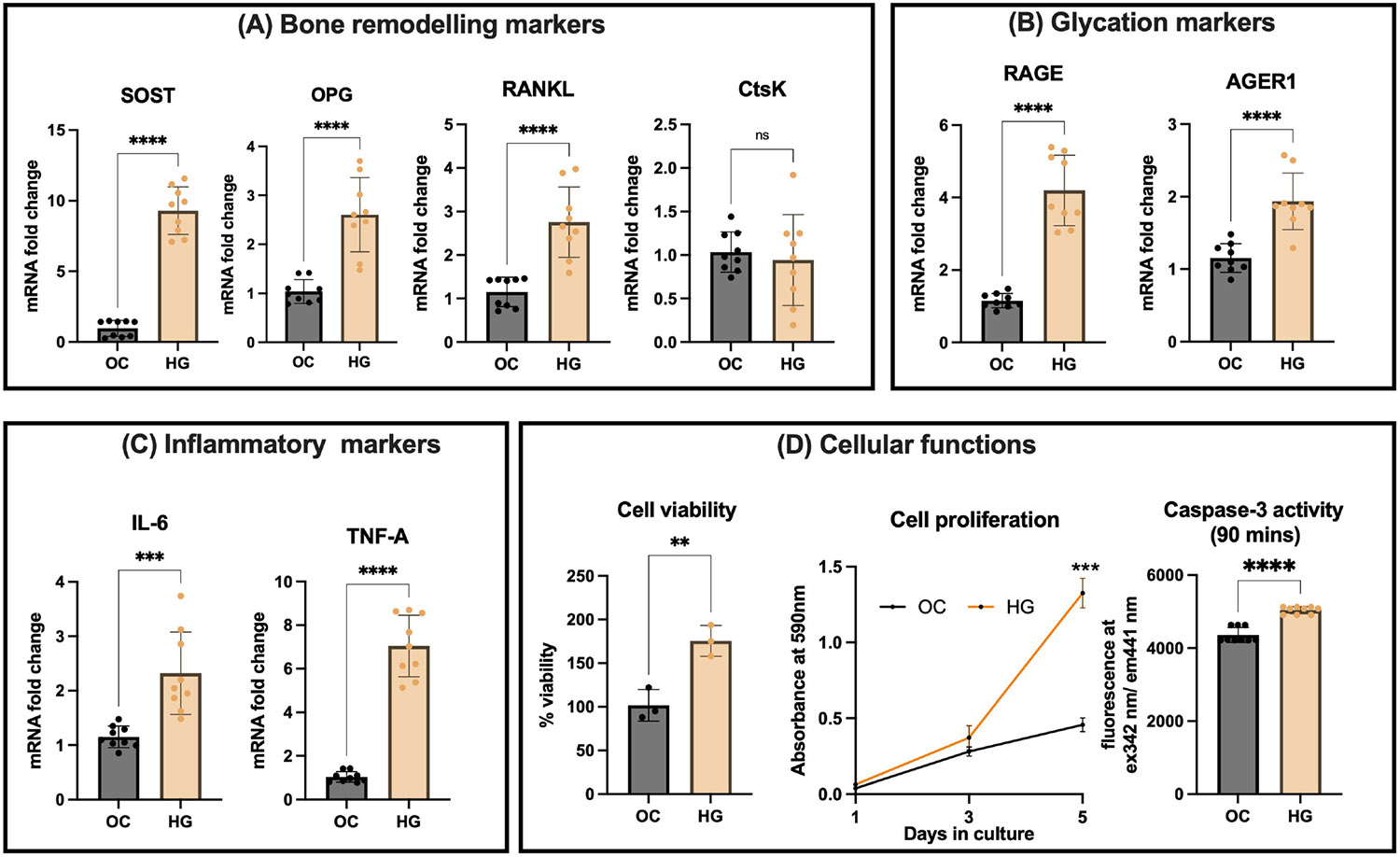
Effects of high glucose (HG) conditions on OCY454-12H osteocyte cells. (A) *Bone remodeling markers*. Significant upregulation is observed in SOST (Sclerostin), OPG (Osteoprotegerin), RANKL (Receptor activator of nuclear factor-*κ*B ligand) mRNA levels under HG conditions compared to osmotic control (OC), suggesting altered bone turnover in response to glucose. No significant change in CTsK (Cathepsin K) expression is observed. (B) *Glycation markers*. Both RAGE (Receptor for Advanced Glycation Endproducts) and AGER1 (Advanced glycated end-product receptor 1) mRNA levels are significantly elevated under HG conditions, indicating increased glycation and potential stress responses in osteocytes. (C) *Inflammatory markers*. Significant increases were observed in IL-6 and TNF-A mRNA levels under HG conditions suggesting an inflammatory response. (D) *Cellular Functions*. Under HG conditions, cells exhibit increased metabolic activity, as well as increased proliferation. Caspase-3 activity is also significantly higher in HG, indicating increased apoptosis.

First, we evaluated the effects of high glucose (HG) conditions, compared to osmotic control (OC) using mannitol, on key markers in mature osteocyte cell line OCY-454-12H. The expression of bone remodeling markers showed significant changes. There was a 10-fold increase in SOST expression under HG (9.3, 95% CI: 8.01 - 10.06, p ≤ 0.0001) compared to OC (0.9, 95% CI: 0.52 - 1.40). There was a 2.4 fold increase in RANKL expression under HG (2.75, 95% CI: 2.13 - 3.38, p ≤ 0.0001) compared to OC (1.15, 95% CI: 0.89 - 1.44). OPG expression increased by 2 fold under HG (2.60, 95% CI: 2.02 - 3.19, p ≤ 0.0001) compared to OC (1.04, 95% CI: 0.85 - 1.22). However, the RANKL/OPG ratio remained unchanged (p = 0.15) in HG conditions. Cathepsin K (*CTsK*) expression was not significantly different between the control (1.03, 95% CI: 0.85 - 1.21) and HG (0.94, 95% CI: 0.54 - 1.35, p = 0.63) (Figure 2A).

Glycation markers exhibited significant upregulation in high glucose environment. RAGE gene expression increased by 3.67 fold in HG (4.20, 95% CI: 3.45 - 4.94, p ≤ 0.0001), compared to OC (1.15, 95% CI: 1.00 - 1.31). AGER1 gene expression was also significantly increased by 1.65 fold in HG (1.94, 95% CI: 1.64 - 2.24, p ≤ 0.0001) compared to OC (1.15, 95% CI: 0.85 - 1.35), suggesting an enhanced AGE-RAGE signaling in HG conditions (Figure 2B).

Inflammatory markers were also elevated in high glucose environment. IL-6 gene expression was increased by 2.02 fold in HG (2.32, 95% CI: 1.74 - 2.90, p ≤ 0.001) compared to OC (1.12, 95% CI: 0.93 - 1.20), whereas TNF-α expression was increased by 7.35 fold in HG (7.04, 95% CI: 5.96 - 8.13, p ≤ 0.0001) compared to OC (1.04, 95% CI: 0.85 - 1.22) (Figure 2C).

Cell viability was significantly higher in HG (176, 95% CI: 131 - 220, p ≤ 0.01) compared to OC (102, 95% CI: 57 - 146). Cell proliferation was significantly higher in HG at Day 5, compared to OC (p = 0.02). Caspase-3 activity was also significantly higher at Day 5 after 90 minutes of HG treatment, suggesting that high glucose environment not only increases cell metabolism but may also enhance apoptotic processes (Figure 2D).

### 3.2. At Physiological Concentrations, CML Selectively Upregulates Glycation Markers, but Does Not Affect Cell Functions, Bone Remodeling, or Inflammatory Markers

**Figure 3.**
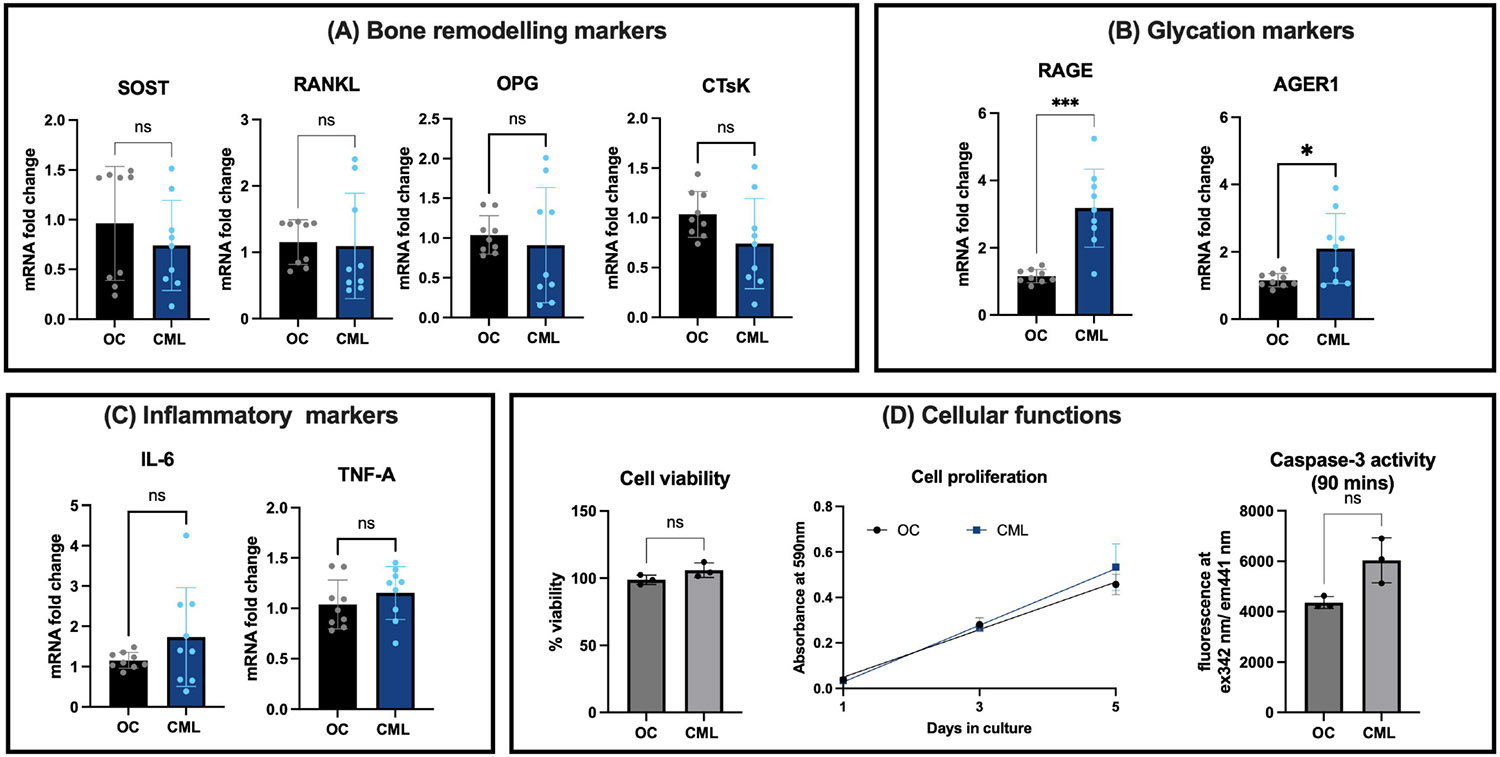
Effects of 3 μM carboxymethyl lysine (CML) treatment on OCY454-12H osteocyte cells. (A) *Bone remodeling markers.* There were no significant differences in the expression of SOST (Sclerostin), OPG (Osteoprotegerin), RANKL (Receptor activator of nuclear factor-*κ*B ligand), and CTsK (Cathepsin K) mRNA levels between CML and osmotic control (OC) groups. (B) *Glycation markers.* Both RAGE (Receptor for Advanced Glycation Endproducts) and AGER1 (Advanced glycated end-product receptor 1) mRNA levels are significantly elevated with CML treatment, indicating increased glycation and oxidative stress responses in osteocytes. (C) *Inflammatory markers.* No significant differences were observed in IL-6 and TNF-A mRNA levels between CML-treated and control groups. (D) *Cellular functions.* Cell viability and caspase-3 activity measurements indicate no significant differences between CML-treated cells and controls, suggesting that at this concentration, CML does not markedly influence cell survival or death processes.

Next, we investigated the effect of 3 μM concentration of carboxymethyl lysine (CML) treatment on osteocytes. The expression of bone remodeling markers in osteocytes showed no significant differences with CML treatment. SOST expression in CML-treated osteocytes was (0.74, 95% CI: 0.39 - 1.09, p = 0.52), similar to that in OC (0.96, 95% CI: 0.51 - 1.41). RANKL showed no significant difference with CML treatment (1.50, 95% CI: 0.48-1.72, p = 0.19) compared to OC (1.15, 95% CI: 0.89 - 1.41). OPG levels were also no different in CML treated cells (0.91, 95% CI: 0.63 - 1.19, p = 0.62) compared to OC (1.04,95% CI: 0.85 - 1.22). CtsK expression was unchanged between CML (0.44, 95% CI: 0.39 - 1.04, p = 0.102) and OC (0.03, 95% CI: 0.85 - 1.21) (Figure 3A).

CML significantly upregulated genes associated with glycation. Specifically, RAGE expression was significantly increased in presence of CML treatment (3.18, 95% CI: 2.29 - 4.07, p ≤ 0.0001) compared to OC (1.15, 95% CI: 1.00 - 1.31, p ≤ 0.001). AGER1 expression also showed a significant increase in CML (2.10, 95% CI: 1.30 - 2.90, p = 0.028) compared to OC (1.14, 95% CI: 0.97 - 1.25), although the extent of change was less pronounced than that of RAGE (Figure 3B).

In terms of inflammatory markers, both IL-6 and TNF-*α* expressions did not exhibit significant changes following CML treatment. IL-6 levels were not significantly different between CML (1.73, 95% CI: 0.99 - 2.47, p = 0.35) versus OC (1.05, 95% CI: 0.80 - 1.25). TNF-*α* levels were also similar between CML (1.15, 95% CI: 0.95 - 1.35, p = 0.35) and OC at (1.04, 95% CI: 0.85 - 1.22) (Figure 3C).

Furthermore, our assessment of cellular functions showed that cell viability and proliferation over a period of 5 days were comparable between the CML-treated groups and controls, indicating that CML does not significantly affect cell survival or proliferation. Additionally, caspase-3 activity, indicative of apoptotic processes, did not show a significant increase following CML treatment (Figure 3D).

These results collectively suggest that at physiological concentrations, CML significantly enhances the expression of glycation markers, particularly RAGE and AGER1. It does not have a significant impact on bone remodeling markers, inflammatory markers, or general cellular functions. This points to a specific effect of CML limited to glycation pathways without inducing cellular toxicity or stress responses under the conditions applied in our study.

## 4. Discussion

Osteocytes play a central role in maintaining skeletal integrity by regulating bone remodeling and responding to metabolic and mechanical signals. However, their responses to diabetic stressors, particularly hyperglycemia and advanced glycation end products (AGEs), remain poorly understood. In this study, we explored the impact of high glucose (HG) conditions and carboxymethyl lysine (CML), a prevalent AGE, on osteocyte-driven pathways associated with bone remodeling, glycation, and inflammation. Our findings reveal distinct effects of HG and CML exposure on osteocytes, providing insights into the molecular mechanisms contributing to diabetes-induced skeletal fragility.

Our data indicate that HG conditions significantly upregulate key markers of bone turnover (SOST, RANKL, OPG), glycation (RAGE, AGER1), and inflammation (IL-6, TNF-*α*), suggesting that hyperglycemia disrupts the balance between bone formation and resorption while promoting oxidative stress and inflammatory responses. Elevated SOST expression under HG is consistent with previous studies demonstrating suppression of the Wnt/*β*-catenin pathway in osteocytes in response to hyperglycemia [69]. Additionally, increased RANKL and OPG expression without a significant change in the RANKL/OPG ratio may indicate a compensatory mechanism aimed at maintaining skeletal homeostasis under hyperglycemic stress [70]. This aligns with recent findings that hyperglycemia-induced oxidative stress in human osteoblast cells simultaneously activates both resorptive and protective pathways [71]. While we did not assess potential glucose metabolism differences between treatment groups, future studies could explore whether osteocytes exhibit metabolic adaptations under prolonged hyperglycemic conditions, which could further influence their response to AGEs. The pronounced upregulation of RAGE and inflammatory markers further underscores the role of AGE-RAGE signaling in promoting oxidative stress and inflammation, which are key drivers of bone fragility in diabetes [32] [35] [39].

Interestingly, the effects of CML exposure differed significantly from those of HG. While CML treatment at physiological concentrations significantly upregulated glycation markers (RAGE and AGER1), it did not affect bone remodeling markers or inflammatory cytokines, nor did it alter cell viability or apoptosis. These results suggest that moderate CML levels primarily engage glycation pathways without triggering downstream inflammatory responses. This observation aligns with prior studies demonstrating that physiological levels of AGEs, including CML [72]-[74], can activate cellular signaling without necessarily inducing cytotoxic effects or inflammatory cascades [75]. For instance, CML has been shown to promote RAGE expression and oxidative stress responses in various cell types, including endothelial cells [76] and fibroblasts [77]. These effects often remain subclinical or non-inflammatory at lower concentrations [8] [63]. Similarly, studies in osteoblasts have reported that AGE exposure enhances RAGE expression and disrupts cellular homeostasis without immediately triggering apoptosis or inflammation [78] [79]. However, there is growing evidence that chronic or high-concentration AGE exposure leads to more pronounced cellular dysfunction, including increased production of reactive oxygen species (ROS), mitochondrial dysfunction, and activation of inflammatory pathways such as NF-*κ*B [80]-[82]. In osteocytes specifically, prolonged AGE exposure has been associated with impaired mechanosensing and altered bone remodeling activity, further exacerbating bone fragility in diabetic conditions [83]. This suggests that while transient or moderate CML exposure may primarily engage glycation pathways, sustained exposure could lead to progressive oxidative damage and inflammatory responses. Long-term exposure to CML may lead to persistent alterations in osteocyte signaling, increasing apoptosis, impairing the lacuno-canalicular network, and further dysregulating bone remodeling by continuously altering RANKL, OPG, and SOST expression. Additionally, prolonged AGE-RAGE signaling is likely to sustain pro-inflammatory cytokine production, such as IL-6 and TNF-*α*, which may contribute to low-grade chronic inflammation and further compromise bone quality. While our current study does not assess these long-term effects, we acknowledge the importance of future investigations that extend the exposure period to determine whether chronic CML treatment could lead to progressive deterioration of osteocyte function and bone integrity. Our findings indicate a threshold effect for CML’s impact on osteocyte function, supporting the need for further research into dose-response dynamics, particularly in the context of chronic diabetic hyperglycemia where AGE accumulation is persistent. A systematic dose-response study would help determine whether higher AGE concentrations or prolonged exposure elicit a threshold-dependent activation of inflammatory and apoptotic pathways, leading to progressive osteocyte dysfunction and compromised bone integrity.

A key limitation of our study is the use of the murine osteocyte cell line OCY454-12H to model diabetic bone pathology. The OCY454-12H cell line was used for this study due to its well characterized mature osteocyte like phenotype, including high Sclerostin expression which is not observed in other osteocyte like cell lines such as MLO-Y4 or IDG-SW3. Although cell lines provide a controlled environment to study molecular pathways, they cannot fully replicate the complexity of *in vivo* bone tissue. *In vivo*, osteocytes are embedded within the mineralized matrix and are subject to mechanical loading and systemic factors, such as hormones and immune cells, influencing their behavior. Therefore, the findings from cell culture studies may not fully reflect the cellular responses in bone tissue under physiological or pathological conditions. Another limitation is that we focused on gene expression analysis rather than protein levels or functional outcomes, which means our findings are based on transcriptional changes and may not fully represent the actual protein expression or post-translational modifications that occur in response to hyperglycemia and CML exposure. This limitation is particularly relevant when interpreting the effects of CML, as protein modifications, such as AGE formation and RAGE activation, could have downstream consequences on protein function, cellular signaling, and osteocyte behavior. Additionally, assessing functional outcomes such as osteocyte apoptosis, lacunar area changes, or bone matrix mineralization under AGE exposure would strengthen the link between molecular alterations and skeletal fragility. Future studies should aim to complement these gene expression data with protein analysis and functional outcomes to provide a more comprehensive understanding of how hyperglycemia and AGEs, such as CML, influence osteocyte function at both the transcriptional and post-translational levels.

Despite these limitations, cell lines remain a valuable tool for investigating cellular responses to specific stimuli, such as hyperglycemia and AGEs, under highly controlled conditions. They allow for precise manipulation of experimental variables, enabling the dissection of molecular mechanisms that would be difficult to isolate *in vivo*. Moreover, the OCY454-12H cell line represents a mature osteocyte phenotype, making it particularly relevant for studying the long-term effects of diabetic conditions on osteocytes, which are the longest-lived bone cells. The use of osteocyte cell lines provides important mechanistic insights into how hyperglycemia and AGEs influence osteocyte function at the molecular level. Our study highlights the potential for targeting the AGE-RAGE axis and oxidative stress pathways as therapeutic strategies to mitigate diabetic bone fragility. The upregulation of SOST under HG conditions suggests that targeting sclerostin, a known inhibitor of bone formation, may help counteract the suppressive effects of hyperglycemia on bone formation. Additionally, the observed increase in both proliferation and caspase-3 activity in HG-treated cells indicates a dual cellular response involving both adaptive and apoptotic mechanisms, which warrants further investigation. Future studies should focus on validating these findings in *in vivo* models to assess the long-term impact of hyperglycemia and AGEs on bone quality and remodeling. Animal models of diabetes would provide critical insights into how chronic hyperglycemia and AGE accumulation affect bone turnover, microarchitecture, and fracture risk over time. Additionally, studies should explore the interaction between different types of AGEs and their cumulative impact on bone tissue, as well as the potential for synergistic effects of HG and AGEs in driving osteocyte dysfunction.

Our findings also raise important questions about the threshold levels of AGEs required to trigger inflammatory and apoptotic responses in osteocytes. Investigating dose-dependent effects of AGEs, including CML, will be critical to understanding how varying glycation burdens influence bone health in diabetic patients. Furthermore, the role of AGER1 as a protective mechanism against AGE-induced damage warrants further exploration. Enhancing AGER1 expression or function may offer a promising therapeutic strategy to counteract the deleterious effects of AGEs on bone cells.

In conclusion, while the use of osteocyte cell lines presents inherent limitations, our study provides valuable insights into the molecular responses of osteocytes to hyperglycemic and AGE stressors. The distinct effects of HG and CML on glycation, inflammation, and bone remodeling markers highlight the complexity of diabetic bone disease. Future research should prioritize *in vivo* studies to validate these findings and investigate potential interventions targeting the AGE-RAGE axis and oxidative stress pathways to mitigate bone fragility in diabetes.
